# High-Throughput Multiplex SARS-CoV-2 IgG Microsphere Immunoassay for Dried Blood Spots: A Public Health Strategy for Enhanced Serosurvey Capacity

**DOI:** 10.1128/spectrum.00134-21

**Published:** 2021-07-28

**Authors:** Linda M. Styer, Rebecca Hoen, Jean Rock, Erica Yauney, Katherine Nemeth, Rachel Bievenue, Monica M. Parker

**Affiliations:** a Wadsworth Center, New York State Department of Health, Albany, New York, USA; b Bureau of Surveillance and Data Systems, New York State Department of Health, Albany, New York, USA; c Department of Biomedical Sciences, School of Public Health, University at Albany, Albany, New York, USA; Memorial Sloan Kettering Cancer Center

**Keywords:** COVID-19, dried blood spot, SARS-CoV-2, immunoassays, serology, serosurvey

## Abstract

Early in the pandemic when diagnostic testing was not widely available, serosurveys played an important role in estimating the prevalence of severe acute respiratory syndrome coronavirus 2 (SARS-CoV-2) in different populations. Dried blood spots (DBS), which can be collected in nonclinical settings, provide a minimally invasive alternative to serum for serosurveys. We developed a Luminex-based SARS-CoV-2 microsphere immunoassay (MIA) for DBS that detects IgG antibodies to nucleocapsid (N) and spike subunit 1 (S1) antigens. The assay uses a 384-well plate format and automated liquid handlers for high-throughput capacity. Specificity was assessed using a large collection of prepandemic DBS and well-characterized sera. Sensitivity was analyzed using serology data from New York State SARS-CoV-2 serosurvey testing and matched diagnostic test results. For DBS, the specificity was 99.5% for the individual N and S1 antigens. Median fluorescence intensity (MFI) values for DBS and paired sera showed a strong positive correlation for N (*R*^2^ = 0.91) and S1 (*R*^2^ = 0.93). Sensitivity, assessed from 1,134 DBS with prior laboratory-confirmed SARS-CoV-2 infection, ranged from 83% at 0 to 20 days to 95% at 61 to 90 days after a positive test. When stratified using coronavirus disease 2019 (COVID-19) symptom data, sensitivity ranged from 90 to 96% for symptomatic and 77 to 91% for asymptomatic individuals. For 8,367 health care workers reporting detailed symptom data, MFI values were significantly higher for all symptom categories. Our results indicate that the SARS-CoV-2 IgG DBS MIA is sensitive, specific, and well-suited for large population-based serosurveys. The ability to readily modify and multiplex antigens is important for ongoing assessment of SARS-CoV-2 antibody responses to emerging variants and vaccines.

**IMPORTANCE** Testing for antibodies to SARS-CoV-2 has been used to estimate the prevalence of COVID-19 in different populations. Seroprevalence studies, or serosurveys, were especially useful during the early phase of the pandemic when diagnostic testing was not widely available, and the resulting seroprevalence estimates played an important role in public health decision making. To achieve meaningful results, antibody tests used for serosurveys should be accurate and accessible to diverse populations. We developed a test that detects antibodies to two different SARS-CoV-2 proteins in dried blood spots (DBS). DBS require only a simple fingerstick and can be collected in nonclinical settings. We conducted a robust validation study and have demonstrated that our test is both sensitive and specific. Furthermore, we demonstrated that our test is suitable for large-scale serosurveys by testing over 56,000 DBS collected in a variety of community-based venues in New York State during the spring of 2020.

On 1 March 2020, the first case of severe acute respiratory syndrome coronavirus 2 (SARS-CoV-2) infection in New York State (NYS) was detected in a symptomatic health care worker (HCW) who had returned to New York City after travel outside the United States. Shortly thereafter, SARS-CoV-2 began spreading rapidly in NYS. Many cases were not confirmed by diagnostic testing, especially those involving asymptomatic individuals who were unaware of their infection and individuals with mild to moderate symptoms who convalesced at home without receiving laboratory confirmation. Considering that only a subset of SARS-CoV-2 infections were reported via diagnostic testing, measuring the seroprevalence of SARS-CoV-2 through antibody testing became an important public health tool for assessing the extent of infections across NYS. Components of an effective SARS-CoV-2 serosurvey include (i) a well-validated, accurate antibody test, (ii) a representative sampling of the target population, including underserved populations, and (iii) a sample size that is large enough to provide sufficient statistical power (1). To meet these criteria, the assay must be sufficiently sensitive and highly specific. Ideally, it is also high-throughput, low-cost, and amenable to rapid deployment in diverse community settings to allow comprehensive population sampling, including vulnerable members of the community.

The use of minimally invasive biospecimen collection methods that are amenable to nonclinical settings can reduce sampling disparities and foster participant diversity. Although serum is the standard specimen for serology testing, collecting blood by venipuncture and transporting blood tubes to a laboratory is often too complex for community and outreach settings (2). Dried blood spots (DBS) collected by fingerstick onto filter paper cards are a viable alternative to serum for serology testing (3). DBS collection requires only minimal training, and transportation of DBS is simplified because they are not considered a biohazard and do not require a cold chain (4). Furthermore, DBS have been shown to be suitable for self/home collection (5–7), another factor that can promote participation in serosurveys.

To address urgent questions regarding the demographic and geographic distribution of SARS-CoV-2 infections in NYS, the New York State Department of Health (NYSDOH) initiated a series of cross-sectional serosurveys of the general community and targeted essential workers between April and June 2020 (8). The strategy included collecting as many as 3,000 DBS samples per day in nontraditional settings like grocery stores and pop-up sites at local community colleges. This necessitated the rapid development, validation, and implementation of a high-throughput assay for detecting SARS-CoV-2 antibodies in DBS samples. To achieve this, we expanded on the Wadsworth Center’s extensive experience in developing Luminex-based microsphere immunoassays (MIAs) (9, 10), including the development of a SARS-CoV-2 pan-Ig MIA for serum (11), and our prior work developing high-throughput assays for detecting IgG antibodies in DBS samples from newborns (12). Building on this cumulative prior experience, we developed a high-throughput immunoassay for detecting SARS-CoV-2 IgG antibodies in DBS. In April 2020, we used an early version of this assay to test 15,101 DBS for reactivity to nucleocapsid (N) protein for a statewide assessment of SARS-CoV-2 seroprevalence in NYS (8). We have since expanded our SARS-CoV-2 IgG DBS assay to detect antibodies to both N and spike subunit 1 (S1) of SARS-CoV-2 and have analyzed data collected on 56,189 DBS samples tested using this multiplex assay.

Here, we describe the performance characteristics of the multiplex SARS-CoV-2 IgG assay for DBS. We augmented laboratory-based validation studies with data collected during the NYSDOH serosurveys and clinical laboratory-reported SARS-CoV-2 diagnostic testing data to systematically define the sensitivity characteristics of the assay. Using median fluorescence intensity (MFI) values produced separately for each antigen, we independently analyzed index values for the N and S1 antigens. We analyzed these semiquantitative antibody results along with coronavirus disease 2019 (COVID-19) symptom data collected from serosurvey participants who had prior, laboratory-confirmed SARS-CoV-2 infection to demonstrate how the assay’s sensitivity differs for asymptomatic and symptomatic individuals.

## RESULTS

### Assay validation.

**FIG 1:**
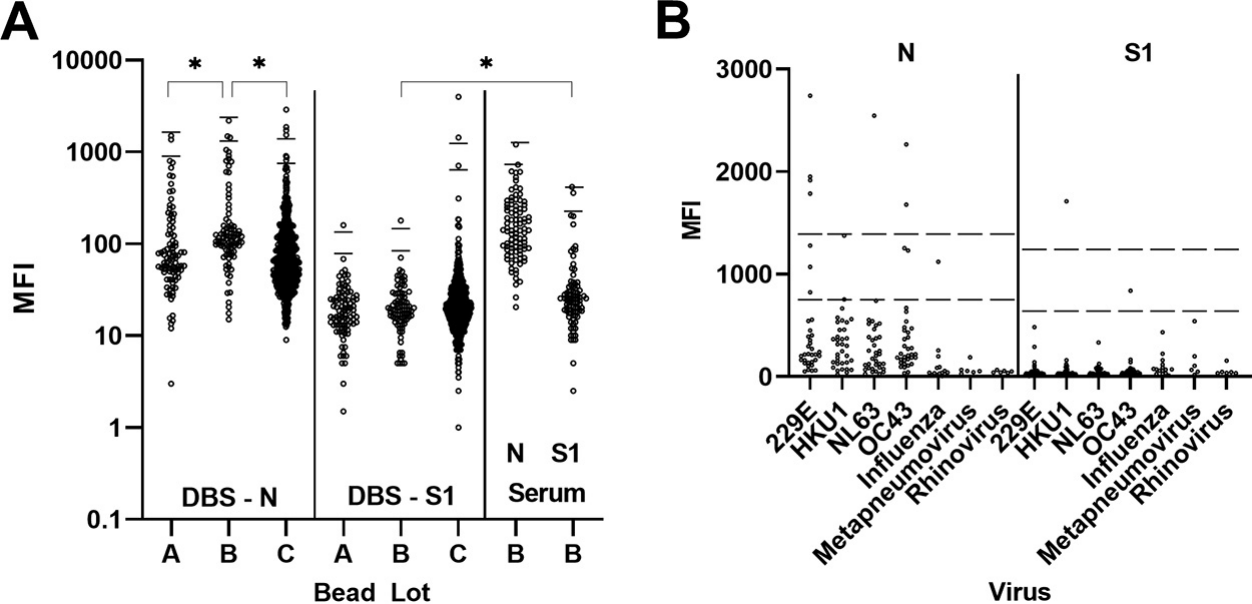
Assay specificity determination. (A) DBS and serum collected prior to December 2019 were tested using three lots of beads for DBS (A, B, C) and one bead lot for serum. *, *P* < 0.005 (B) Serum specimens containing antibodies to respiratory viruses, including other human coronaviruses, were tested. Lower line is cutoff for indeterminate results; upper line is cutoff for reactive results. MFI, median fluorescence intensity; N, nucleocapsid; S1, spike subunit 1.

To determine cutoff values and assay specificity, we tested 730 DBS and 701 serum specimens collected in NYS prior to December 2019 on three lots of beads coupled to SARS-COV-2 N and S1 antigens (Fig. 1A). Significant differences occurred between median MFI values for N-coupled bead lots but not S-coupled lots when they were tested using DBS. Bead lot B was tested on both DBS and serum. MFI values were higher in serum than in DBS for S beads but were similar for N beads. Cutoffs for each bead lot were set at the mean MFI + 6 SD to be classified as reactive. Results that fell between the mean MFI + 3 SD and mean MFI + 6 SD were classified as indeterminate, and below the mean MFI + 3 SD were nonreactive. Cutoff values for S bead lot C were higher than for lots A and B because lot C was tested on a larger set of DBS (*n* = 547) than lots A and B (*n* = 92 and 91, respectively). Serum containing antibodies to other respiratory pathogens, including other human coronaviruses, were tested to assess cross-reactivity (Fig. 1B).

In DBS, the specificity was 99.5% for each individual bead set. For the combined results from both bead sets, the specificities were 98.9% for the N or S1 criteria and 100% for the N and S1 criteria. In serum, specificity was lower in the respiratory panel than in normal serum, especially for the N bead set (95.8% in respiratory versus 99.6% in normal serum). Specificity remained at 100% using the combined N and S1 criteria (Table 1).

**TABLE 1:** Number of reactive samples and specificity of individual and combined antigens for DBS and serum

Antigen bead set	Normal DBS (*n* = 730)		Normal serum (*n* = 701)		Respiratory panel serum (*n* = 167)	
	Reactive (No.)	Specificity (%)	Reactive (No.)	Specificity (%)	Reactive (No.)	Specificity (%)
N	4	99.5	3	99.6	7	95.8
S1	4	99.5	2	99.7	1	99.4
N or S1	8	98.9	5	99.3	8	95.2
N and S1	0	100.0	0	100.0	0	100.0

### Concordance studies.

**FIG 2:**
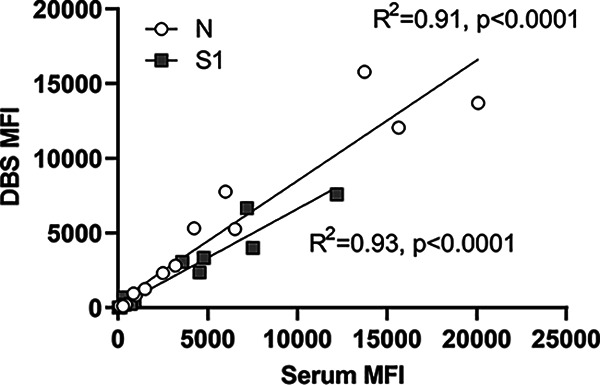
Analysis of paired serum and laboratory-prepared DBS. MFI, median fluorescence intensity; N, nucleocapsid; S1, spike subunit 1.

**FIG 3:**
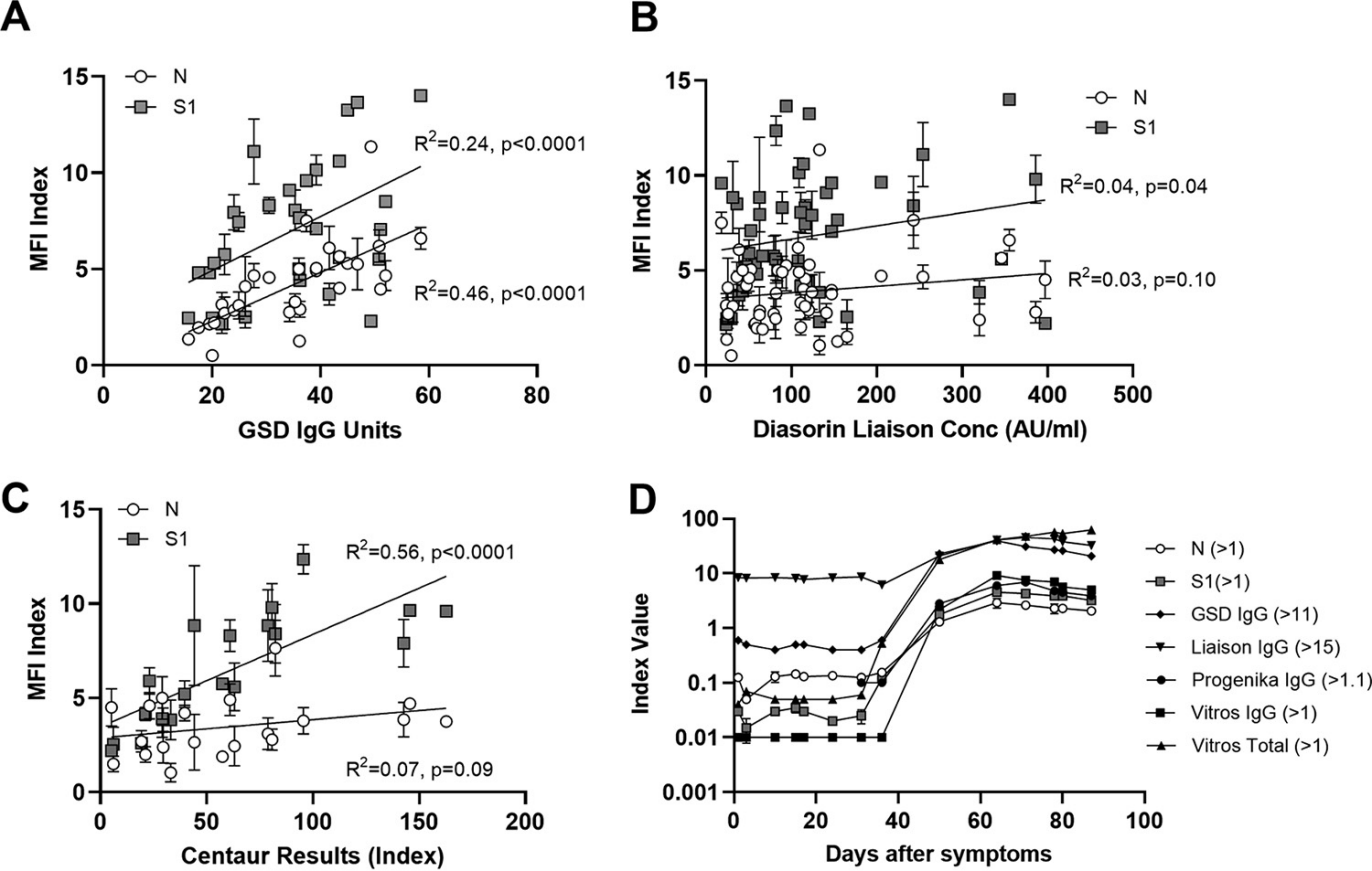
Concordance with commercially available SARS-CoV-2 immunoassays. Specimen panels from Access Biologicals were tested, and MFI index values for nucleocapsid (N) and spike subunit 1 (S1) were compared to results from other commercial assays as provided by the panel provider. (A) Thirty sera with comparator results from GSD SARS-CoV-2 IgG (Gold Standard Diagnostics). (B) Fifty sera with comparator results from Liaison SARS-CoV-2 S1/S2 IgG (Diasorin). (C) Twenty sera with comparator results from Advia Centaur SARS-CoV-2 total (Siemens). (D) Twenty-eight plasma samples collected from a single individual between days 1 and 98 after COVID-19 symptom onset. The reactive cutoff values for each test are listed next to the name.

To demonstrate the correlation between DBS and serum in our assay, we tested paired serum and spiked DBS specimens. MFI values for DBS and serum showed a strong positive correlation for both N (*R*^2^ = 0.91) and S1 (*R*^2^ = 0.93) (Fig. 2). To assess concordance between our assay and other SARS-CoV-2 immunoassays, we tested commercially available serum panels collected from individuals with confirmed COVID-19 infections and compared the results with those reported by the supplier for two assays with emergency use authorization (EUA) from the U.S. Food and Drug Administration (FDA) and one CE-marked assay (Fig. 3A to C). All 50 samples were reactive on our SARS-CoV-2 IgG assay (on either the N or S1 bead sets), matching reactive results on the comparator assays. The MFI index values of N and S1 bead sets were significantly correlated with Gold Standard Diagnostics IgG units (N, *R*^2^ = 0.46, *P* < 0.0001; S, *R*^2^ = 0.24, *P* < 0.0001). The Centaur results were significantly correlated with the S1 MFI index (S, *R*^2^ = 0.56, *P* < 0.0001). We also tested a commercially available seroconversion panel in which 28 plasma samples were collected from the same person on various days after COVID-19 infection. The results were compared with the index values of five FDA EUA or CE-marked assays (Fig. 3D). All assays were nonreactive for samples collected between 1 and 36 days after symptom onset. At the next time point, day 50 after symptom onset, all assays became reactive.

### Sensitivity analysis.

**FIG 4:**
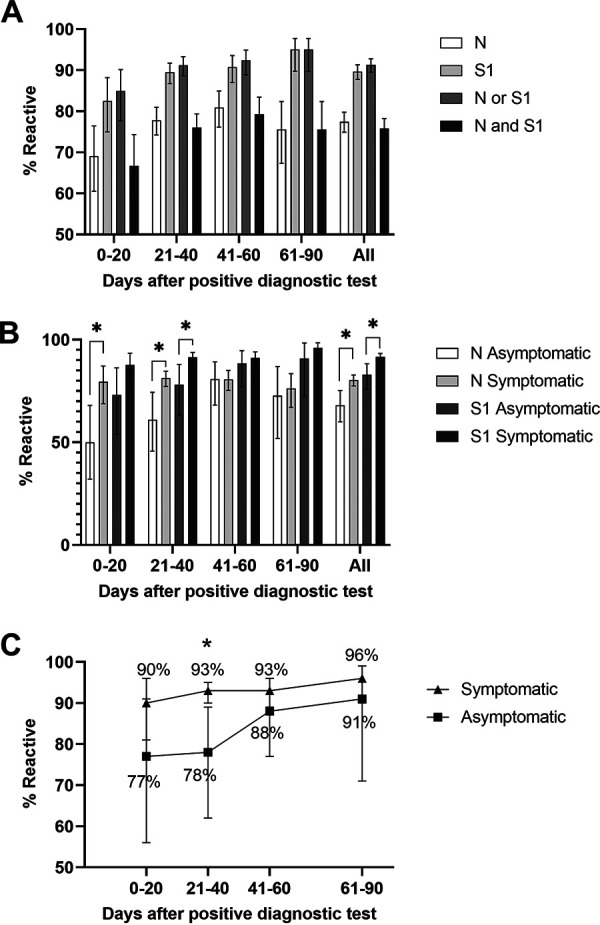
Sensitivity analysis on specimens with prior laboratory-confirmed SARS-CoV-2 infection. (A) Reactivity for individual nucleocapsid (N) and spike (S1) bead sets and reactivity based on “OR” and “AND” result criteria by days after positive diagnostic test. (B) Reactivity for N and S1 bead sets by days after positive diagnostic test and presence/absence of COVID-19 symptoms. (C) Overall assay reactivity (reactive = N or S1 reactive) by days after positive diagnostic test and presence/absence of symptoms. Error bars = 95% CI; *, 0.01 < *P* < 0.05.

We analyzed test data from DBS collected from 56,189 individuals during the statewide serosurvey. After merging serosurvey data with laboratory-reported SARS-CoV-2 diagnostic test data from the New York State surveillance database, we identified 1,134 samples that were collected from individuals with a confirmed positive SARS-CoV-2 diagnostic test result prior to DBS collection (Table 2). The specimen collection dates for the positive diagnostic laboratory tests were available for all 1,134 members of this group. We used this date and the date of DBS collection to calculate the number of days between infection and antibody testing and analyzed reactivity against the number of days after a positive diagnostic test. Although the actual onset of infection may have been several days prior to diagnostic sample collection, this analysis provides a reasonable estimate of the assay’s sensitivity relative to time from infection. Reactivity to S1 (range, 83 to 95%) was significantly higher than reactivity to N (range, 69 to 81%) at 21 to 40, 41 to 60, and 61 to 90 days after a positive diagnostic test. Reactivity to either N or S1 did not increase significantly by days after positive diagnostic test (Fig. 4A). At all time points, the highest sensitivity was achieved when reactivity to either N or S1 was used to classify a specimen as seropositive. Although requiring reactivity to both N and S1 resulted in 100% specificity, sensitivity was reduced to below 80% at all time points.

**TABLE 2:** Characteristics of individuals with a positive SARS-CoV-2 diagnostic test prior to DBS collection

Characteristic	No. (%) who reported:			
	Symptoms (*n* = 847)	No symptoms (*n* = 141)	Unknown (*n* = 146)	Total (*n* = 1,134)
Sex				
Male	323 (38)	74 (52)	114 (78)	511 (45)
Female	524 (62)	67 (48)	18 (12)	609 (54)
None provided	0 (0)	0 (0)	14 (10)	14 (1)
Ethnicity				
Hispanic or Latino	254 (30)	50 (35)	73 (16)	327 (29)
Not Hispanic or Latino	578 (68)	88 (62)	72 (49)	738 (65)
None provided	15 (2)	3 (2)	51 (35)	69 (6)
Race				
White	350 (41)	40 (28)	64 (44)	454 (40)
Black/African American	157 (19)	40 (28)	13 (9)	210 (19)
Asian	126 (15)	18 (13)	4 (3)	148 (13)
Multiracial/other	198 (23)	39 (28)	0 (0)	237 (21)
None provided	16 (2)	4 (3)	65 (45)	85 (7)
Age (yr)				
18–34	189 (22)	47 (33)	53 (36)	289 (25)
35–44	197 (23)	24 (17)	49 (34)	270 (24)
45–54	204 (24)	26 (18)	28 (19)	258 (23)
55–64	200 (24)	26 (18)	16 (11)	242 (21)
65+	57 (7)	18 (13)	0 (0)	75 (7)

Individuals with a positive diagnostic test were classified as asymptomatic or symptomatic based on their answer to a question in the serosurvey intake form asking if they experienced COVID-19 symptoms (Table 2). Antibody reactivity to N was significantly lower in asymptomatic individuals than in symptomatic individuals at 0 to 20 and 21 to 40 days after a positive diagnostic test (Fig. 4B). S1 reactivity was also significantly lower in asymptomatic individuals than in symptomatic individuals at 21 to 40 days after a positive diagnostic test. Reactivity to S1 was significantly higher than reactivity to N in symptomatic individuals at 21 to 40, 41 to 60, and 61 to 90 days after a positive diagnostic result. Overall assay reactivity, which was based on reactivity to N or S, increased from 90 to 96% among symptomatic and 77 to 91% among asymptomatic individuals from 0 to 90 days after a positive diagnostic test. At days 21 to 40, overall assay reactivity was significantly higher among symptomatic than among asymptomatic individuals (Fig. 4C).

### Analysis of reactivity versus symptoms.

**FIG 5:**
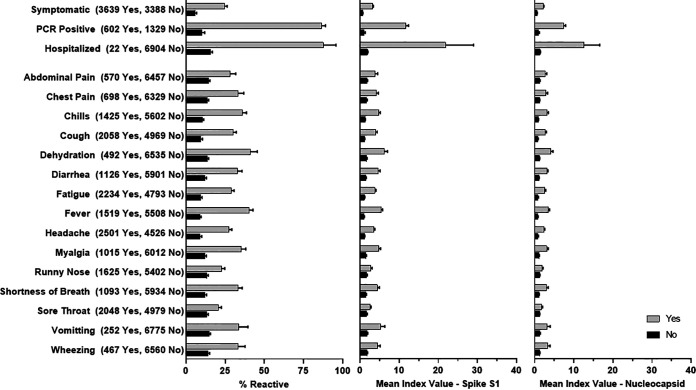
Percent reactive and mean index values for spike S1 and nucleocapsid by symptom category for health care workers. The numbers who reported “yes” and “no” for each symptom are listed. Error bars indicate 95% confidence intervals.

For all serosurvey participants who reported on their COVID-19 symptom experience, there was a statistical association between experiencing symptoms and having a reactive antibody test result. Among 39,458 participants asked about COVID-19 symptoms, 55.6% (*n* = 2,294) of 4,122 reactive individuals reported experiencing symptoms compared to 22.4% (*n* = 7,912) of 35,356 non-reactive individuals who reported symptoms (*P* < 0.0001). Of the cohorts that participated in the serosurveys, the only group for which detailed information about specific COVID-19 symptoms was collected was the health care worker (HCW) cohort (*n* = 8,367). Across all symptom categories, the percentages of reactive and mean MFI index values for S1 and N were significantly higher among those who reported experiencing the symptom than for those who did not (Fig. 5). In addition to collecting specific symptom data, the HCW questionnaire included questions about the severity of illness, including information about hospitalization and SARS-CoV-2 PCR test results. Consistent with the analysis using laboratory-reported diagnostic test data (Fig. 4), a self-reported positive diagnostic test and more severe illness were associated with statistically higher mean MFI index values (Fig. 5). The largest recorded mean N MFI index, 12.5 (95% CI, 8.5 to 16.6), was seen in those who reported being hospitalized for COVID, compared to 1.4 (95% CI, 1.3 to 1.5) among those who were not hospitalized. Similarly, the mean S1 MFI index values for hospitalized and nonhospitalized HCWs were 21.8 and 1.9, respectively.

## DISCUSSION

To assist with NYS’s public health response to the COVID-19 pandemic, we developed a high-throughput immunoassay that is both sensitive and specific for detection of SARS-CoV-2 IgG antibodies in DBS samples. A strength of our study is the data from >1,100 individuals with confirmed SARS-CoV-2 infection prior to DBS collection, including dates of laboratory diagnosis and self-reported presence or absence of COVID-19 symptoms. This analysis showed that for both N and S1, sensitivity increased with the time from a positive diagnostic test, but S1 was significantly more sensitive than N for detecting SARS-CoV-2 antibodies at all time points. Using criteria in which reactivity to either the N or S1 antigen classifies a sample as seropositive provides the greatest sensitivity while maintaining a 99% specificity. For applications where maximal specificity is needed, defining seropositivity based on a reactivity result for both N and S1 raises the specificity to 100%; however, the sensitivity will be reduced. Our data also show that the assay’s sensitivity is higher when testing people who experienced COVID-19 symptoms than for those who were asymptomatic, and this difference was significant for samples collected 21 to 40 days after a positive diagnostic test. This relationship between symptoms and antibody response is consistent with data from other studies (13–15). These data highlight the importance of using well-characterized samples from a heterogeneous population that includes asymptomatic as well as symptomatic individuals to obtain an accurate sensitivity assessment of SARS-CoV-2 serology assays.

The suitability of DBS for SARS-CoV-2 serology has been reported (5, 6, 16–18); however, the multiplexing capacity of Luminex technology combined with the high-throughput capacity of our 384-well plate format makes our DBS assay particularly well suited for assessing population-based seroprevalence. Using a single 3.2-mm DBS punch, we can assess reactivity to multiple antigen-coupled bead sets simultaneously in a single well and produce separate, semiquantitative results for each antigen. Validation of a flow-cytometer-based multiplex microsphere immunoassay (MMIA) for DBS was recently described, and while this MMIA is likely to be suitable for large-scale SARS-CoV-2 serosurveys, the application of the assay in a serosurvey was limited to 264 participants (16). We have demonstrated sustained high-throughput use of our SARS-CoV-2 MIA by testing 56,189 DBS over an 8-week period in the spring of 2020. During that period, our assay was performed manually and each technologist was able to manually test 696 samples in an 8-h shift. Our capacity has since increased to 2,784 samples per day by incorporating two automated liquid handlers into the protocol.

The ease with which antigen-coupled bead sets can be added and removed is especially useful in rapidly evolving outbreak situations. The ability to multiplex and simultaneously obtain separate measurements of antibody reactivity for different antigens has proven valuable. As the pandemic began to surge in NYS, we relied on an existing supply of SARS-CoV N protein, prepared in-house during the 2003 SARS outbreak, to develop an initial SARS-CoV-2 immunoassay (8, 11). As SARS-CoV-2 antigens became commercially available, we were able to multiplex new and old bead sets in the same test well, which allowed us to continue testing samples while concurrently validating new antigens. The ability to independently measure antibodies to different antigens has additional implications as the COVID-19 vaccination phase unfolds. We are currently modifying our multiplex SARS-CoV-2 assay by incorporating additional antigens and standards to enhance its ability to distinguish antibody responses due to natural infection from those induced by vaccination.

Health outcome disparities have been observed during the COVID-19 pandemic (19), and the importance of adequately representing population diversity, including underserved and vulnerable members, in COVID-related research and surveillance has been emphasized. This includes serosurveys, which provide valuable data on disease prevalence, as well as insight into the characteristics of antibody response and potential immunity within the population. By limiting studies to specimens collected in clinical settings, many important populations may be excluded, leading to gaps in scientific knowledge (1). DBS provide a minimally invasive sample collection method that is amenable to nonclinical settings (3). Public health programs have turned to DBS testing in community settings as a means to increase infectious disease testing in underserved and vulnerable populations (20–22). During the NYS SARS-CoV-2 serosurveys, fingerstick DBS samples were collected from a demographically diverse group of individuals in a variety of settings, including community-based pop-up sites, grocery stores, and community college gymnasiums. Although the DBS samples tested in our serosurvey were collected by trained individuals, self-collection of DBS at one’s home has been demonstrated to be a feasible method of obtaining samples for COVID-19 serosurveys (5–7).

Our high-throughput SARS-CoV-2 IgG MIA offers many advantages for obtaining large-scale seroprevalence data through community sampling; however, some limitations should be noted. Each antigen exhibits low-level cross-reactivity with other coronaviruses. False reactivity can be largely eliminated if reactivity to both N and S1 is required for seropositivity, but this may reduce sensitivity, especially for detection of asymptomatic cases. Although MFI values are considered proportional to antibody reactivity, the assay is only validated for reporting qualitative results. While serology assays can be calibrated by generating standard curves from monoclonal antibodies, this method is prone to variability and quantitative values will not be consistent between methods. The first WHO International Standard of anti-SARS-CoV-2 immunoglobulin recently became available from the National Institute for Biological Standards and Control. We intend to incorporate this calibration standard into our assay and transition to reporting quantitative SARS-CoV-2 IgG results. This will allow us to assess the variation in antibody levels detected using our assay and will allow for a more meaningful comparison of results across studies. Our current method indicates reactivity to S1, but we currently do not have a method to further characterize functionality, such as neutralization capacity, with DBS samples. However, the development of serological methods to characterize the neutralizing capacity of SARS-CoV-2 antibodies using DBS is under way.

In conclusion, we have developed a high-throughput, multiplex SARS-CoV-2 IgG immunoassay for DBS with well-defined performance characteristics. This assay was deployed at large scale during a period of surging SARS-CoV-2 infections in New York State, where the use of fingerstick-collected DBS was the key to a rapid, representative sampling of the population that was needed to help inform public health decision making. The multiplexing capacity of this system, which allows rapid modification of assay components, was critical during the early pandemic response period when reagents were limited, and it continues to be valuable as we contend with new viral variants and assessment of vaccine response.

## MATERIALS AND METHODS

### Specimens.

Dried blood spots (DBS) collected by fingerstick and submitted to the Wadsworth Center for clinical testing before December 2019 were used to determine background reactivity and assay specificity. Mock DBS samples used in validation studies and as positive controls were created by centrifuging SARS-CoV-2 antibody-negative EDTA whole blood, removing the plasma, and adding an equal volume of SARS-CoV-2 IgG-positive serum or plasma to the blood cells. After mixing, 50 μl of spiked blood was spotted onto Whatman 903 filter paper and dried at room temperature for 4 h. Fingerstick-collected DBS were collected as previously described (8) from serosurvey participants at pop-up sites, grocery stores, health care facilities, and educational institutions in NYS between 17 April and 12 June 2020 and transported at ambient temperature to the Wadsworth Center Laboratory (Albany, NY) by courier. All participants were at least 18 years old and provided general consent for SARS-CoV-2 IgG testing. Serum specimens shared with us by William Lee included specimens (i) submitted to the Wadsworth Center for clinical testing, (ii) collected in New York from healthy individuals prior to December 2019 at the New York Blood Center and the American Red Cross, and (iii) collected at Weill Cornell Medical Center and Columbia University Medical Center following molecular testing-confirmed respiratory infections, as well as (iv) sera confirmed as positive for antibodies to non-SARS-CoV-2 human coronaviruses obtained from Regeneron (Tarrytown, NY). Commercial serum panels (COVID-19 30-member panel, COVID-19 20-member panel, and COVID-19 seroconversion panel) were obtained from Access Biologicals (Vista, CA).

### Data collection.

Data were analyzed from testing conducted on DBS collected from the (i) general public sampled at grocery stores (19 April to 28 April 2020 and 9 June to 12 June 2020), (ii) health care workers (17 April to 4 June 2020), (iii) NYC Fire Department and Police Department employees (27 April 2020), (iv) New York State Police (1 May to 4 May 2020), (v) NYS civil service employees designated essential (8 May to 18 June 2020), (vi) food service workers (8 May to 6 June 2020), (vii) grocery store workers (11 May to 6 June 2020) and (viii) pharmacy workers (23 May to 5 June 2020). The New York State COVID-19 Antibody Testing System (NYSCATS), a Microsoft Dynamics 365 customer relationship management (CRM) application developed by the NYSDOH and Microsoft Corporation, was used to collect participant data, schedule antibody testing, and report results. For samples collected from 24 April to 1 May 2020, data were first collected using Microsoft Excel spreadsheets and then loaded into the NYSCATS system once it was formatted and cleaned. Personal identifying information and demographic data were collected on all participants, including region of residence, age, gender, race, and ethnicity. Customized questionnaires within the NYSCATS application were used to collect additional data, including COVID-19 testing history, symptoms, and disease severity. These questionnaires changed over time and varied by cohort, and thus, not all information is available across all tested cohorts. Serosurvey participant data were exported from NYSCATS and merged with clinical laboratory-reported SARS-CoV-2 diagnostic testing data from the NYSDOH Electronic Clinical Laboratory Reporting System (ECLRS) database (https://www.health.ny.gov/professionals/reportable_diseases/eclrs). The match was completed based on the last name, first name, and date of birth of those with serological data in NYSCATS to retrieve ECLRS SARS-CoV-2 diagnostic testing results and collection dates for serosurvey participants. After matching and deduplicating the merged NYSCATS/ECLRS data set, individual records were deidentified for data analysis.

### Assay procedure.

Magplex-C microspheres (Luminex Corp., Austin, TX) with different bead regions were coupled to SARS-CoV-2 nucleocapsid (N) and spike subunit S1 (S1) antigens (Sino Biological, Wayne, PA). Microspheres were washed using activation buffer (0.1 M monosodium phosphate, pH 6.2) and activated by adding 50 mg/ml sulfo-NHS (*N*-hydroxysulfosuccinimide) and EDC [1-ethyl-3-(3-dimethylaminopropyl) carbodiimide hydrochloride] (Thermo Scientific Pierce). Beads were then incubated with antigen at a concentration of 5 μg antigen/1 × 10^6^ beads in coupling buffer [0.5 M 2-(*N*-morpholino) ethanesulfonic acid, pH 5.0]. Coupled beads were diluted in storage buffer (phosphate-buffered saline [PBS] with 1% bovine serum albumin [BSA], 0.02% Tween 20, 0.05% azide, pH 7.4) to a concentration of 1 × 10^6^ beads/ml.

A 3.2-mm DBS punch was added to 250 μl elution buffer (Tris-buffered saline, 1% casein blocker) (Bio-Rad Laboratories, Hercules, CA) in round-bottom, nontreated, polystyrene 96-well plates at room temperature (19°C to 22°C) for 1 h. Eluate (25 μl) was transferred to nonbinding 384-well plates (Greiner Bio-One, Monroe, NC) along with 25 μl of beads (1,250 beads/bead set/well). Eluate and beads were incubated together for 30 min at 37°C with shaking (300 rpm) in the dark. Samples were washed three times using wash buffer (PBS, 2% BSA, 0.02% Tween 20, 0.05% azide, pH 7.5) on a BioTek 405 TSUS microplate washer. After washing, samples were incubated with 50 μl phycoerythrin-tagged goat-anti human IgG (Invitrogen, Thermo Fisher) in the dark at 37°C and 300 rpm for 30 min. Plates were washed as described above. Beads were resuspended in 90 μl xMap sheath fluid (Luminex Corp., Austin, TX) and incubated for 1 min at room temperature at 300 rpm in the dark. Serum specimens were tested as described above except that serum was diluted 1:101 in PBN buffer (PBS, 1% BSA, 0.05% sodium azide, pH 7.4) and 25 μl was used in the assay. Samples were analyzed using a FlexMap 3D instrument (Luminex Corp., Austin, TX), which produces a median fluorescence intensity (MFI) for each bead set. Based on validation and optimization studies, the mean MFI of at least 92 negative DBS was used to set cutoffs. Each bead set is evaluated separately. Results that are less than the mean MFI + 3 standard deviations (SD) were classified as nonreactive, between mean MFI + 3 and + 6 SD is indeterminate, and greater than the mean MFI + 6 SD is considered reactive. The MFI index was calculated for each bead set by dividing the MFI value by the reactive cutoff value; values of >1.0 indicate a reactive result for that bead set. Reactivity was determined separately for each bead set and for both bead sets, with “N or S1” defined by having a reactive result for either the N or S1 bead set and “N and S1” defined by having a reactive result for both the N and S1 bead sets.

### Data analyses.

Analysis of antibody testing results, NYSCATS data, and ECLRS matched data was conducted using SAS 9.4. Basic frequency analysis was conducted to describe each of the cohorts and the supplemental questionnaire data collected for each cohort. The distribution of SARS-CoV-2 antibody reactivity was also analyzed by demographics, the timing of sample collection, and symptomology when possible. Data from persons with positive diagnostic tests prior to the collection of samples for the antibody test were used to assess test sensitivity. At the time of analysis, it was not possible to definitively distinguish the diagnostic test method from the surveillance data. During the period of this analysis (17 April to 18 June 2020), the vast majority of diagnostic testing was conducted using PCR-based methods; however, it is possible that some reported diagnostic test results were from antigen tests. Analysis of test performance data was conducted in GraphPad Prism. Bead lot analysis was performed using Kruskal-Wallis and Dunn’s multiple-comparison test or the Mann-Whitney test. Linear regression was used to assess concordance between DBS and serum and between different assays. A chi-square test and 95% confidence intervals (CIs) were used to assess differences in proportions. Approval for human subject research was obtained from the New York State Department of Health Institutional Review Board (protocols 10-002 and 20-021).
